# An Unusual Presentation of Neonatal Pyloric Stenosis: A Case Report

**DOI:** 10.7759/cureus.97735

**Published:** 2025-11-25

**Authors:** Omar Ismail, Zaki Ghorayeb, Ghina Saade, Carla El Haber

**Affiliations:** 1 Department of Pediatrics, Faculty of Medicine, Hotel-Dieu de France University Medical Center, Saint Joseph University, Beirut, LBN; 2 Department of Pediatric Surgery, Faculty of Medicine, Hotel-Dieu de France University Medical Center, Saint Joseph University, Beirut, LBN; 3 Department of Neonatology, Faculty of Medicine, Hotel-Dieu de France University Medical Center, Saint Joseph University, Beirut, LBN

**Keywords:** gastrointestinal obstruction, hypertrophic pyloric stenosis, neonatal vomiting, typical age range, unusual presentation

## Abstract

Neonatal vomiting is a nonspecific symptom with a broad range of possible causes, including systemic illness, metabolic disturbances, and gastrointestinal obstruction. One important surgical condition is hypertrophic pyloric stenosis, which usually manifests within the first few months of life with persistent, forceful vomiting and metabolic imbalance. This report describes a rare case of the condition occurring in a three-day-old neonate, underscoring the importance of timely diagnosis and early surgical management to avoid complications.

## Introduction

Neonatal vomiting is a common and nonspecific symptom that may reflect a broad spectrum of underlying disorders, including infections, metabolic derangements, sepsis, or gastrointestinal obstruction [1]. Hypertrophic pyloric stenosis (HPS) is among the most common surgical causes of nonbilious, projectile vomiting in early infancy [2]. In this condition, hypertrophy of the pyloric muscle leads to gastric outlet obstruction [3]. If untreated, it may result in dehydration, electrolyte disturbances, and metabolic disturbances [4]. Early recognition is critical for timely intervention and optimal outcomes [5].

## Case presentation

**Figure 1:**
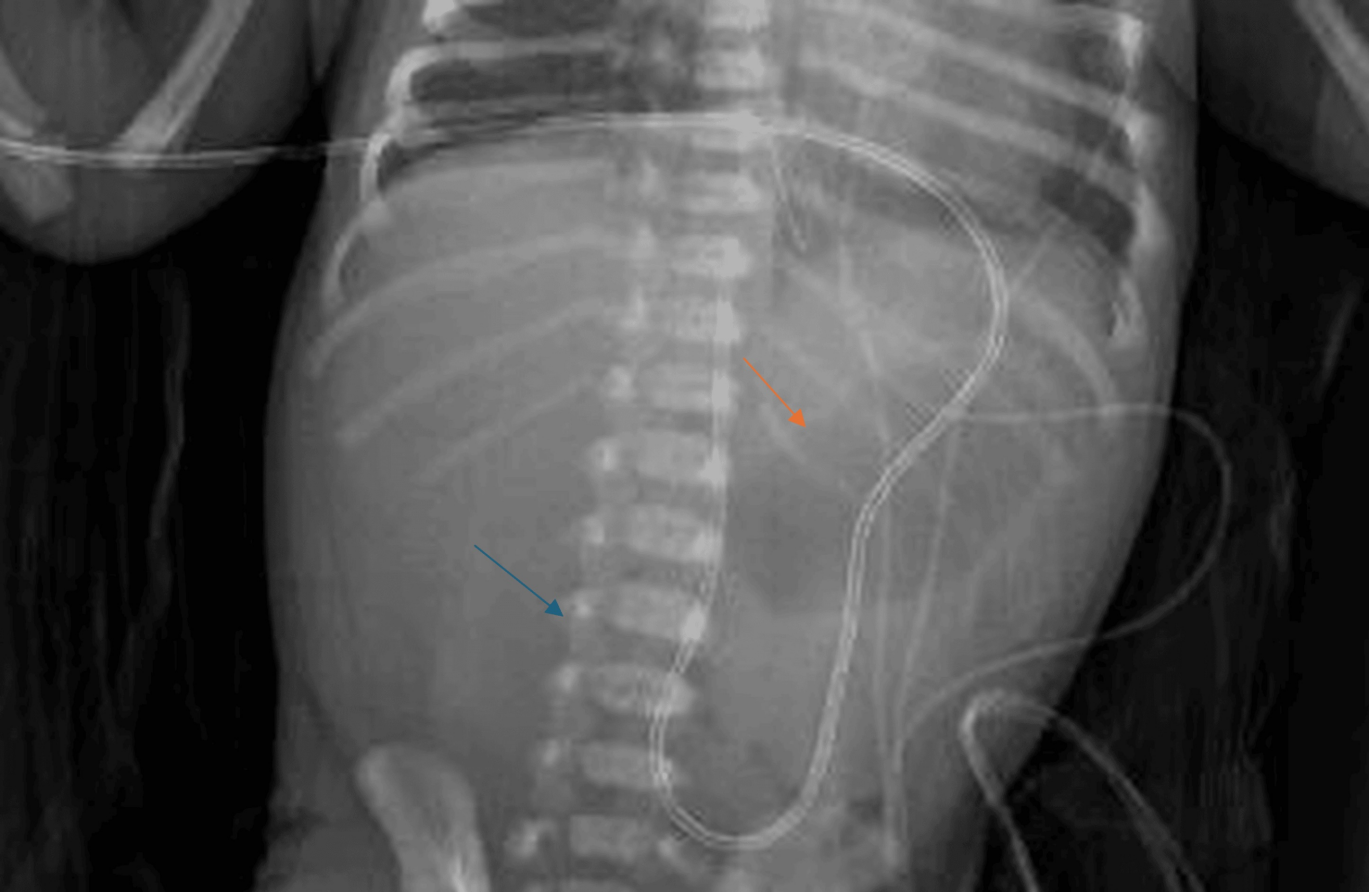
Abdominal X-ray showing abnormal bowel gas pattern The orange arrow points to the prominent gastric gas bubble (dilated stomach), and the blue arrow points to an area of the abdomen with paucity of distal bowel gas

A 36-week gestational age male neonate, weighing 2,970 g at birth, was delivered via C-section due to placenta previa, to a 29-year-old mother (G1P1A0), a former smoker with an otherwise unremarkable medical history. The delivery was uncomplicated, and the infant received routine care in the nursery, passing urine and meconium within the first 24 hours. Regurgitations were noted since birth; however, persistent nonbilious emesis began on day 3 of life, accompanied by mild abdominal distension with normal bowel sounds and no palpable mass on examination. Initial sepsis workup was conducted. Electrolytes were normal. Intravenous antibiotics and hydration were initiated, and the patient remained nil per os with a nasogastric tube on free drainage. Abdominal X-ray showed an abnormal gas pattern (Figure 1).

**Figure 2:**
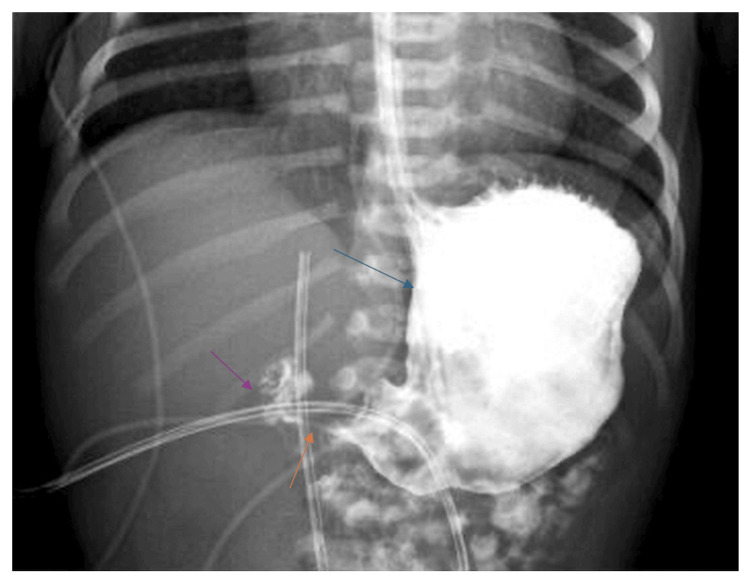
Delayed gastric emptying and intermittent threadlike passage of the contrast at the antral level The blue arrow points to the large, contrast-filled structure in the left upper abdomen. This is indeed the distended stomach, which is the expected appearance in pyloric obstruction. The orange arrow points to a very thin, trickling column of contrast at the gastric outlet. This matches what is typically described as the “string sign,” representing contrast passing through a narrowed pyloric canal. The purple arrow points just distal to the pylorus, where almost no contrast is visible, consistent with paucity of contrast beyond the pylorus, a hallmark of hypertrophic pyloric stenosis

An upper gastrointestinal series revealed delayed gastric emptying with intermittent threadlike passage of the contrast at the antral level (Figure 2).

**Figure 3:**
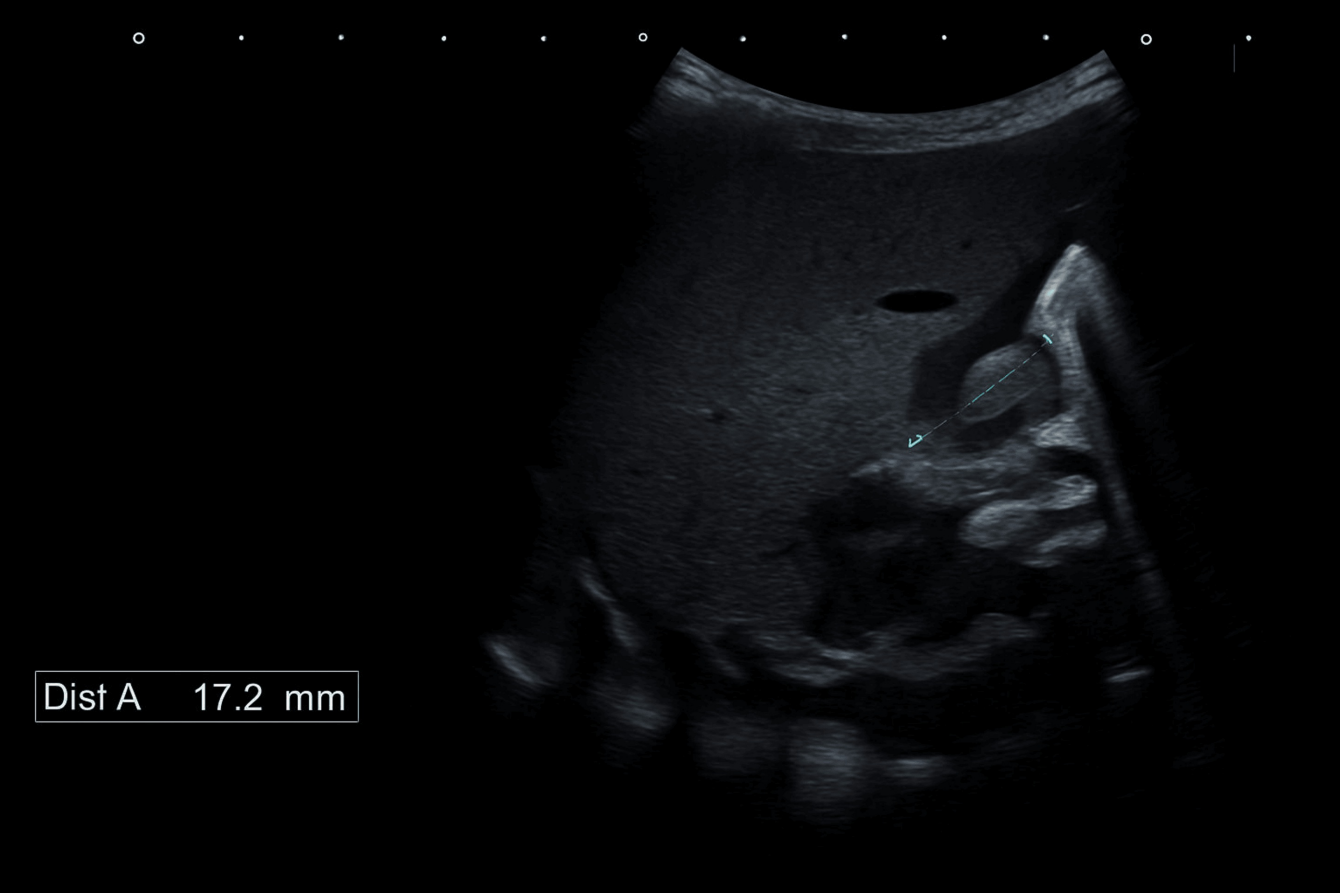
Ultrasonography showed a canal length of 17.2 mm

**Figure 4:**
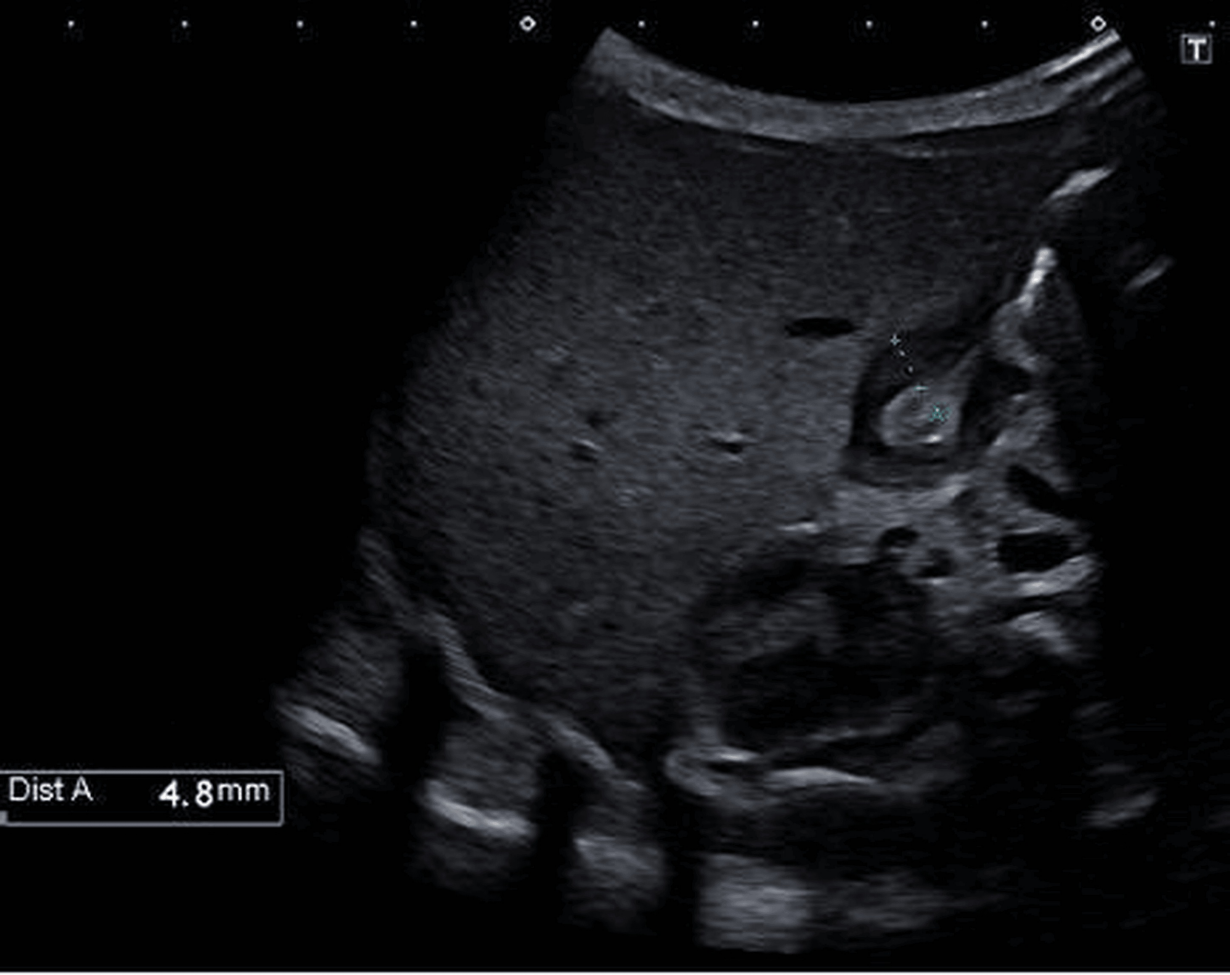
Ultrasonography showing a pyloric muscle thickness of 4.8 mm

Ultrasonography showed features consistent with HPS, with a pyloric muscle thickness of 4.8 mm and a canal length of 17.2 mm (Figures 3, 4).

An exploratory laparotomy was performed on day 7 of age, confirming the diagnosis of HPS. A pyloromyotomy was successfully completed to correct the obstruction. Feeding was well tolerated postoperatively, and the baby was discharged two days after the surgery.

## Discussion

Pyloric stenosis (HPS) occurs in 2-3.5 per 1,000 live births [6]. The typical presentation is between three weeks and three months of life [7]. HPS does not usually present within the first week of age, as in this case. Risk factors include prematurity, positive family history of pyloric stenosis, male gender, firstborn children, maternal smoking, and early exposure to erythromycin [7-13]. Early presentation of pyloric stenosis, occurring during the first seven days of life, makes the diagnosis very challenging, as vomiting often suggests overfeeding or reflux. However, conditions like sepsis, necrotizing enterocolitis, or obstructive lesions of the gastrointestinal tract must be ruled out.

Pyloric stenosis commonly manifests as forceful, nonbilious vomiting, typically occurring soon after feeding. Affected infants often remain hungry and eager to feed again, a phenomenon known as the “hungry spitter.” A firm, olive-shaped mass may be palpable in the right upper quadrant, and gastric peristaltic waves can sometimes be observed before vomiting. If vomiting persists over time, it can lead to hypokalemic and hypochloremic metabolic alkalosis [14]. Our case lacked the projectile nature of vomiting and the electrolyte disturbances.

Abdominal ultrasound is the diagnostic modality of choice, with key criteria including pyloric muscle thickness >4 mm and canal length >18 mm [15]. Immediate treatment involves correcting dehydration and metabolic disturbances, followed by surgical correction of the stenosis.

## Conclusions

This case report highlights the importance of considering pyloric stenosis even in neonates outside the typical age range. Early diagnosis and intervention are essential to prevent complications and ensure favorable outcomes. In this case, the neonate underwent timely surgical management, leading to rapid resolution of vomiting and normalization of hydration and electrolyte levels. The patient recovered well without postoperative complications and was discharged in stable condition. Follow-up assessments confirmed appropriate weight gain and normal feeding tolerance, indicating sustained clinical improvement. This case underscores that with prompt recognition and definitive treatment, even atypical presentations of pyloric stenosis can achieve excellent long-term outcomes.
